# Therapeutic Differentiation of Tumor-derived Insulin-producing Cells Selected for Resistance to Diabetogenic Drugs

**DOI:** 10.1155/EDR.2000.233

**Published:** 2000

**Authors:** Konstantin Bloch, Pnina Vardi

**Affiliations:** 1 Felsenstein Medical Research Center Tel-Aviv University Beilinson Campus Petah Tikva 49202 Israel

## Abstract

Differentiation therapy has been proposed as a new approach to selectively engage the process of tumor cell differentiation during chemotherapy of cancer. Our recent *in vitro* study suggests that such an approach can be extended and utilized for the selection of tumor-derived insulin-producing cells for transplantation. Repeated treatment with streptozotocin selected toxin resistant subpopulation of insulin producing tumor RINmS cells, characterized increased level of insulin content and secretion. In the present study RINmS cells were found to have higher glucose sensitivity and insulin response compared with parental RINm cells. In addition, compounds known to induce elevated level of cAMP beta-cells, such as isobutyl methyl xanthine, and forskolin, potentiated glucose-induced insulin secretion of RINmS, but had no effect on the naive parental RINm cells. These experiments suggest that differentiation therapy can be utilized for engineering insulin producing cells with improved defense and secretory mechanisms.


*Int. Jnl. Experimental Diab. Res.*, Vol. 1, pp. 233–237
Reprints available directly from the publisher
Photocopying permitted by license only

© 2000 OPA (Overseas Publishers Association) N.V.
Published by license under
the Harwood Academic Publishers imprint,
part of The Gordon and Breach Publishing Group.
Printed in Malaysia.

### Therapeutic Differentiation of Tumor-derived Insulin-producing Cells Selected for Resistance to Diabetogenic Drugs

KONSTANTIN BLOCH* and PNINA VARDI†

*Felsenstein Medical Research Center, Tel-Aviv University, Beilinson Campus, Petah Tikva 49202, Israel*

*(Received 3 April 2000; In final form 4 May 2000)*

**Differentiation therapy has been proposed as a new approach to selectively engage the process of tumor cell differentiation during chemotherapy of cancer. Our recent *in vitro* study suggests that such an approach can be extended and utilized for the selection of tumor-derived insulin-producing cells for transplantation. Repeated treatment with streptozotocin selected toxin resistant subpopulation of insulin producing tumor RINmS cells, characterized by increased level of insulin content and secretion. In the present study RINmS cells were found to have higher glucose sensitivity and insulin response compared with parental RINm cells. In addition, compounds known to induce elevated level of cAMP in beta-cells, such as isobutyl methyl xanthine, and forskolin, potentiated glucose-induced insulin secretion of RINmS, but had no effect on the naive parental RINm cells. These experiments suggest that differentiation therapy can be utilized for engineering insulin producing cells with improved defense and secretory mechanisms.**

*Keywords*: Streptozotocin resistance; Cell differentiation; RIN cells; Insulin; Secretagogues

*Abbreviations*: STZ, streptozotocin; AL, alloxan; RINmS, streptozotocin-selected RINm cells; GLUT-2, glucose transporter 2

### INTRODUCTION

Tumor derived insulin-producing beta-cell lines constitute a potential source for islet transplantation.[1,2] To achieve long-term functioning, transplanted beta cells must posses a high level of differentiation to allow adequate glucose sensing, insulin processing and secretion, and an effective defense mechanism.

Recently, differentiation therapy has been proposed as a new approach to selectively engage the process of tumor cell differentiation during chemotherapy of cancer.[3] According to this approach, cytotoxic agents, including chemical compounds and cytokines used in cancer therapy, can induce drug resistance, but in certain conditions, can also lead to elimination of tumorigenic cells and to recovery of normal cell homeostasis.[4–6]

We have recently found that repeated, *in vitro* treatment, with streptozotocin (STZ), an anti-neoplastic compound utilized for the treatment of

*Corresponding author. Fax: 972-3-9211478, Tel: 972-3-9376280.
†e-mail: pvardi@post.tau.ac.il patients with insulinoma, selects toxin resistant subpopulation of insulin-producing RINmS tumor cells.[7,8] The higher resistance to both STZ and alloxan (AL), was found to be at least partially dependent on the lower expression of the glucose transporter 2 (GLUT-2) in the selected cells. Despite the lower expression of GLUT-2 in the resistant cells, their functional capacity, reflected by the increased intracellular insulin content and insulin release, did not seem to be impaired. In the present study we further evaluated the insulin response to different secretagogues, and used the proliferation capacity of RINmS cells as a marker of differentiation stage.

### METHODS

### Cell Culture and Selection Procedure

The RINm cells, which have been previously described in detail,[9] were cultured in RPMI 1640 medium, supplemented with 10% FCS, 2 mmol/l L-glutamine, 100 IU penicillin and 100 μg/ml streptomycin. Cells were grown in plastic tissue culture flasks at 37°C in 95% humidified air with 5% $CO_2$. The toxin resistant cells (RINmS) were developed by exposing cells to 10 mM of STZ in 2 passages, as previously described.[8] Parallel cultures of untreated RINm cells were used as controls. The cells (passages 14–22), free of micoplasma contamination, were used in all experiments.

### Insulin Response to Increasing Glucose Concentrations and Effect of IBMX and FSK on Insulin Release

Prior to the experiments (72 h), $2 \times 10^5$ cells/well were seeded in 24-well plates. After washing, cells were preincubated for 1 h in glucose-free Krebs–Ringer bicarbonate buffer (KRB) and reincubated in the buffer at 37°C for additional

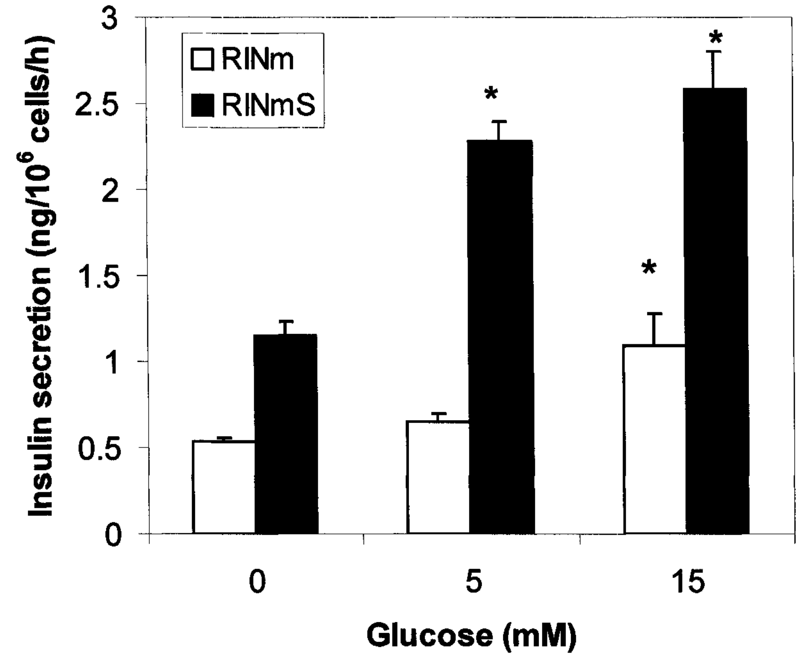

FIGURE 1 Glucose-induced insulin secretion from RINm and RINmS cells. Effect of various glucose concentrations was assessed over a 1 h incubation period at 37°C. The level of insulin was estimated by radioimmunoassay. Values are mean ± SE from 4 independent experiments. *$p < 0.05$ compared with basal secretion (0 mM of glucose).

hour with various secretagogues: glucose 5 and 15 mM; isobutyl methyl xanthine (IBMX) 0.1 mM; forskolin (FSK) 1 μM. The level of basal insulin secretion into KRB was evaluated in the absence of secretagogues. Supernatants were centrifuged and collected for insulin determination by RIA.

### Insulin Radioimmunoassay

The insulin content was determined by RIA using a rat insulin standard. Samples were thawed and centrifuged prior to insulin determination.

### Cell Growth Characteristics

Cells were seeded into 24-well plates in culture medium at a cell density of $2 \times 10^5$/well. Following trypsinization, the cell number per well was determined under a light microscope in a counting chamber at 2, 3, 6 and 8 days after seeding.

### Statistical Analysis

Analysis of variance (ANOVA) was utilized for evaluation of the statistical significance of differences between groups. Results are presented as

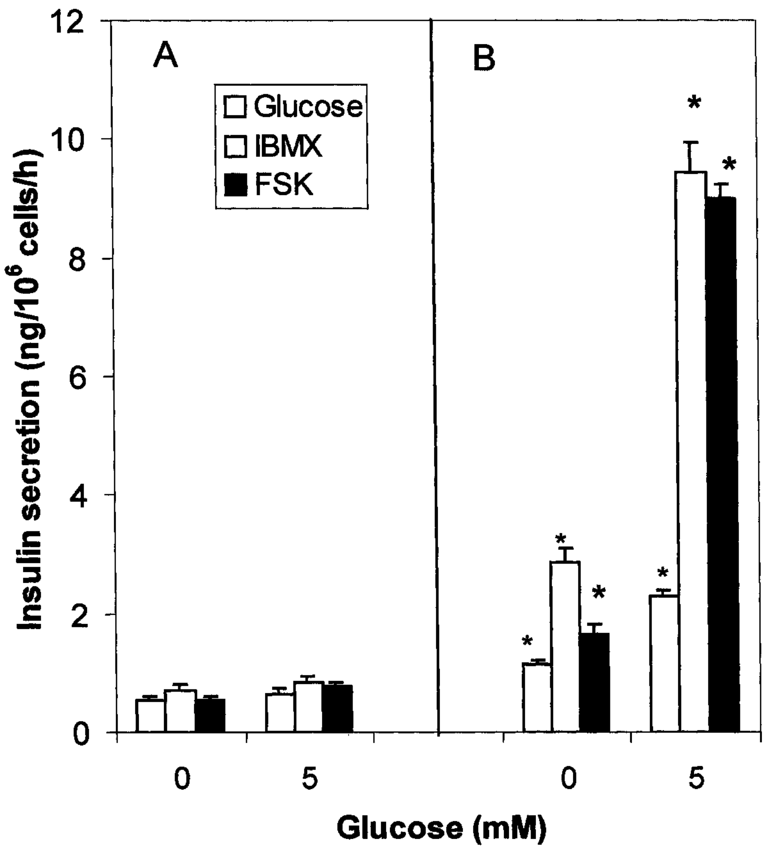

FIGURE 2 Insulin secretion from RINm (A) and RINmS (B) cells in response to glucose and IBMX or FSK. Three days before incubation. $4 \times 10^5$ cells/well were seeded in 12-well plates. After two washes, cells were preincubated for 60 min in glucose-free buffer and incubated for another 60 min in the same buffer containing the various additions (glucose 0 mM, 5 mM; IBMX 0.1 mM; FSK 1 μM). The results represent the mean of 4 separate experiments. $^*p < 0.05$ compared with RINm cells under the same conditions.

mean values ± SEM of 3–4 independent, repeated experiments. Experiments were performed in triplicate; $p < 0.05$ was considered significant.

### RESULTS AND DISCUSSION

We previously demonstrated that repeated exposure of tumor-derived insulin-producing RINm cells to high doses of STZ leads to selection of a cell subpopulation (RINmS) with a higher resistance not only to STZ, but also to AL.[7,8] The higher cell defense property of the surviving cells was found to be associated with a decreased expression of GLUT-2, which acts as a transporter of STZ and AL. In addition to their modified defense potential, resistant RINmS cells were found also to contain and release significantly higher level of insulin when compared with their untreated parental RINm cells. The finding of low GLUT-2 expression in the highly resistant and more differentiated RINmS cells is unexpected and opposes that of high level of GLUT-2 and extreme cell sensitivity to diabetogenic toxins reported in experiments with rodent islets. Such controversy could result from the different cell pathways of natural selection in the whole animals, and the artificially-induced *in vitro* selection of RINmS cells.

We further evaluated the cell functional capacity and found that RINmS cells are more sensitive to increasing concentrations of glucose when compared with RINm cells. Thus, incubation with 5 mM of glucose alone induced a twofold increase in insulin secretion in RINmS and had no effect in RINm cells (Fig. 1). Furthermore, IBMX and FSK, compounds which induce elevation of cAMP level in beta-cells, lead to a 4-fold increase of glucose (5 mM) - induced insulin secretion in the toxin-resistant RINmS but not in the parental cells (Fig. 2). These data suggest that the lower GLUT-2 expression of RINmS cells, previously reported,[8]

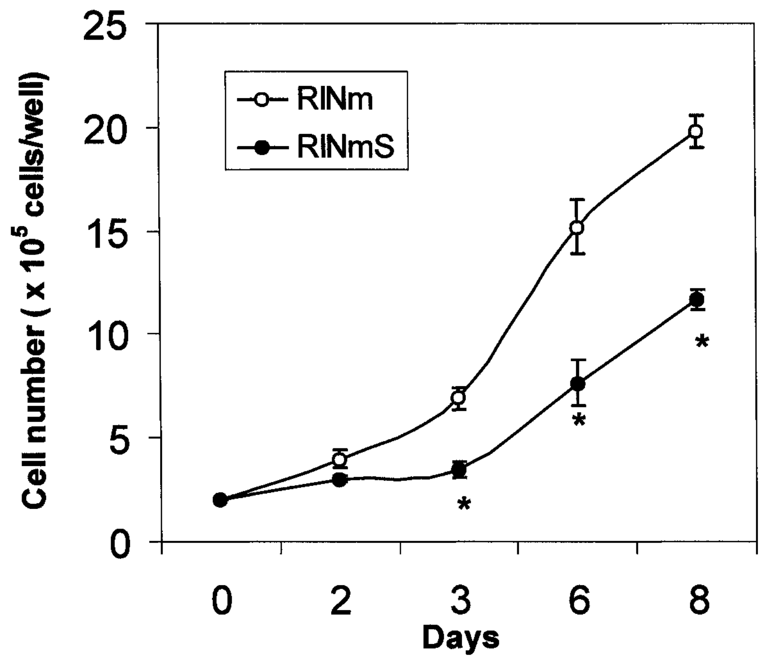

FIGURE 3 Growth curves of RINm (open circles) and RINmS (solid circles) cells expressed as number of cells per well at 2, 3, 6 and 8 days after seeding. Data are given as means ± SEM from 3 independent experiments. *$p < 0.05$ compared with RINm cells.

is probably sufficient to maintain the mechanism of glucose induced insulin secretion. It is possible that the higher cellular insulin content of RINmS cells, its improved regulation and secretion is probably due to transition of this cell population to a higher cell differentiation stage. This possibility is further supported by our finding of lower cell proliferative activity in RINmS when compared with parental RINm cells (Fig. 3). A similar induction of drug resistance in association with cell differentiation and growth suppression was previously reported in selected tumor cells following antineoplastic drug treatment.[3,5] Over-expression of multi drug resistance genes (*mdr*1), and decreased expression of nuclear transcription factor genes (*c-myc*), which control tumorigenic development are believed to be the underlying molecular mechanisms which activate such cellular pathway.[10] We speculate that the alkylating agent STZ, not only induces resistance to diabetogenic drugs, but also induces increased DNA methylation, which in turn reinforces differentiation process in selected cells.

### Acknowledgment

This work was partially supported by grant #6774195 of the Israeli Ministry of Science and Art.

### References

[1] Newgard, C. B. (1994). Cellular engineering and gene therapy strategies for insulin replacement in diabetes, *Diabetes*, **43**, 341–350.

[2] Efrat, S. (1998). Cell-based therapy for insulin-dependent diabetes mellitus, *Eur. J. Endocrinol.*, **138**, 129–133.

[3] Waxman, S., Borden, E. C., Lotan, R., Levens, D. and Young, C. W. (1993). Differentiation therapy of cancer: laboratory and clinical investigations, *Cancer Res.*, **53**, 4109–4115.

[4] Jetten, A. M., Nervi, C. and Vollberg, T. M. (1992). Control of squamous differentiation in tracheobronchial and epidermal epithelial cells: role of retinoids. *J. Natl. Cancer Inst. Monogr.*, **13**, 93–100.

[5] Prados, J., Melguizo, C., Marchal, J. A., Velez, C., Alvarez, L. and Aranega, A. (1998). Therapeutic differentiation in a human rhabdomyosarcoma cell line selected for resistance to actinomycin D. *Int. J. Cancer*, **75**, 379–383.

[6] Marchal, J. A., Prados, J., Melguizo, C., Gomes, J. A., Campos, J., Gallo, M. A., Espinosa, A., Arena, N. and Aragenda, A. (1999). GR-891: a novel 5-fluorouracil acyclonucleoside prodrug for differentiation therapy in rhabdomyosarcoma cells. *Br. J. Cancer*, **79**, 807–813.

[7] Bloch, K. and Vardi, P. (1996). Streptozotocin-induced selection of insulin enriched cells in a heterogeneous population of RINm cells. *Diabetologia*, **39** (Suppl. 1), 72.

[8] Bloch, K., Zemel, R., Bloch, O., Grief, H. and Vardi, P. (2000). Streptozotocin and alloxan-based selection improves toxin resistance of insulin-producing RINm cells. *International J. Experimental of Diabetes Research* (in press).

[9] Gazdar, A. F., Chick, W. L., Oie, H. K., Sims, H. L., King, D. L., Weir, G. C. and Lauris, V. (1980). Continuous, clonal, insulin- and somatostatin-secreting cell lines established from a transplantable rat islet cell tumor, *Proc. Natl. Acad. Sci. USA*, **77**, 3519–3523.

[10] Prados, J., Melguizo, C., Fernandez, A., Aranega, A. E., Alvarez, L. and Aranega, A. (1995). Inverse expression of *mdr* 1 and *c-myc* genes in a rhabdomyosarcoma cell line resistant to actinomycin D. *J. Pathol.*, **180**, 85–89.

